# The Royal Army Medical Corps Section

**Published:** 1911-11-25

**Authors:** 


### THE ROYAL ARMY MEDICAL CORPS SECTION.

### The British Red Cross Society and its Voluntary Aid Detachments.

The work of raising Voluntary Aid detachments for service with the Territorial Medical Service, undertaken by the British Red Cross Society at the request of the military authorities, has been already noticed in The Hospital. As the movement has been in existence for some considerable time, it may serve a useful purpose to review briefly the present position, and to make clear the relation of the British Red Cross Society to the work concerned. Dealing first with the results already attained, they must be regarded as very creditable and satisfactory. Excellent progress has been made in carrying out the scheme by the Red Cross County Committees. A very large number of the counties have given a hearty and patriotic response to the appeal made to them, and notably is this so in the case of the counties of Gloucester, Essex, Devon, Derbyshire, and Glamorgan. Over five hundred detachments, with a personnel of 15,000, have been already raised in the United Kingdom, and at January 31 of this year no fewer than four hundred and fifteen of these detachments, with a personnel of 12,475, have been numbered and registered by the War Office. In the matter of training everything is being done by the different county directors to ensure the efficiency of the members of the detachments in the first-aid, nursing, and cooking duties required from them, while opportunities for practical work have been afforded them in several instances in the shape of demonstrations in the field, as at Oakham, an account of which was given in The Hospital for December 10, 1910. In view of this we consider there is every reason to believe that the detachments will be found serviceable should they be called upon in the event of invasion.

Passing now to the position of the British Red Cross Society in relation to the work undertaken, matters are somewhat confused. Primarily, no doubt, the Territorial County Associations are charged with the responsibility of the organisation of the Voluntary Aid detachments in connection with the Territorial Force of their respective counties, but in Paragraph 15 of the War Office scheme for the organisation of Voluntary Aid in England and Wales they are empowered, and also recommended, to delegate the formation and organisation of the detachments to the British Red Cross Society, the military authorities further adding that that body is the only one the War Office is prepared to recognise for the purpose. Notwithstanding, however, this very clear indication of the course to be pursued by the Territorial Associations, permission has also been given to them to employ other means of raising the detachments, the only proviso being that the War Office will hold the associations responsible and can take no cognisance of these other outside agencies.

The outcome of this arrangement is that the action of the Territorial Associations has varied throughout England and Wales. No doubt a large number of the associations have rallied round the British Red Cross Society and have handed over to that body the organisation of Voluntary Aid in their respective counties, in accordance with the War Office recommendation, but others have undertaken to carry out the provisions of the scheme themselves, and others have delegated the work to one or more responsible bodies. One association has even gone the length of accepting detachments which are "raised either through some parent organisation or as independent units." To have such a varied and disconnected channel of supply for its Voluntary Aid to the Territorial Force does not, we confess, recommend itself to us as being a satisfactory plan of organisation. This is not, it must be remembered, the question of the instruction of the detachments. In that we have always advocated a wide latitude and have supported the view that any certificate in first-aid and nursing that has a recognised value should admit its holder to membership of a detachment. In the matter, however, of the organisation of these Voluntary Aid detachments, we hold just as strongly that there should be unity and homogeneity, otherwise there will arise overlapping and friction, and there will be lost that cohesion that leads to smooth, harmonious, and efficient working in the day of trial. We confess we think the arrangement come to in Scotland, where all the Territorial Associations have handed over the provision of Voluntary Aid to the Scottish branch of the British Red Cross Society, and where the detachments are all grouped for administrative purposes into a Scottish Territorial Red Cross Brigade, has a good deal to recommend it. The brigade is under the management of the Scottish Red Cross branch, and through the latter all communications must pass, and with that branch the War Office deals. There is thus a central and guiding authority and the detachments all work under the same rules and conditions of training and service.

The need of central authority in the matter of this scheme of Voluntary Aid detachments is being well illustrated at the present time by the recent action of the presidents of some of the county branches of the British Red Cross Society. Holding a series of meetings, they passed at them certain resolutions, two of which they sent direct to the Council of County Territorial Associations. The first one was to the effect that, as under War Office regulations Voluntary Aid detachments have now become part of the Technical Reserve, a capitation grant should be made annually to each detachment that satisfactorily passes its official inspection. The second resolution asked that an assurance should be given to all members of the Voluntary Aid detachments by the War Office that on mobilisation, or on being called out in aid of, or by the Government, they will be placed on the same footing as the Royal Army Medical Corps as regards pay, rations, travelling expenses, and compassionate allowances. We express no opinion on the importance or otherwise of the subject-matter of these two resolutions beyond observing that if a capitation grant is to be paid by the Government, the military authorities will certainly limit to the smallest size possible the number of detachments and personnel enrolled. It is not likely that they will lay themselves open to unlimited demands upon the public purse, nor will money be paid without additional restrictions accompanying it, so that in all probability the objects desired may have a tendency to affect the future of the detachments, restricting them in numbers and depriving them to some extent of their voluntary and patriotic character. At the Territorial Council meeting, Sir Richard Temple, speaking for the St. John Ambulance Association, felt convinced that they would be absolutely opposed to asking for any financial assistance from the War Office for any detachments they may have raised.

We understand that at a subsequent meeting of the St. John Ambulance Association it was resolved that the ambulance department of the Order objects to any capitation grant being given to any of its Voluntary Aid detachments and does not consider such a grant necessary. What we feel very strongly about this step that has been taken by some of the County Red Cross Committees is that the irregular way in which the matter has been carried through constitutes a very unfortunate course of action and establishes a bad precedent. There was nothing to prevent the presidents of County Red Cross branches meeting and passing resolutions, but these should have been sent direct to the Council of the British Red Cross Society for their consideration, and their permission obtained before distributing them not only to presidents and directors in England, but also to the Scottish Council and all the Scottish branches. Even Lord Dartmouth, who laid resolutions before the Council of County Territorial Associations, felt that his action was premature and that it was important to be sure that what were the views of a few counties represented those of the majority. Consequently, it was decided to send the two resolutions to all the Territorial Associations asking for their opinion, and adding a note to the effect that the St. John Ambulance detachments did not wish for any grants.

There the matter rests at present, but the incident is instructive and should not be lost upon the Council and Executive of the British Red Cross Society. Through their agency there has been called into existence a large body of useful and necessary workers for aid to the sick and wounded Territorial soldiers in case of invasion. It rests with the Society to see that detachments are placed under an organisation that will enable them to work smoothly and harmoniously in peace as well as in war, and that all suggestions for their modification or improvement should pass through the proper channel.

